# Supplementary material for: Multiple pals gene modules control a balance between immunity and development in Caenorhabditis elegans
Source: bioRxiv. 2023 Jan 18:2023.01.15.524171. Preprint. [Version 1] doi: 10.1101/2023.01.15.524171 (PMC9882112; doi:10.1101/2023.01.15.524171)
Supplement: 1 [file NIHPP2023.01.15.524171v1-supplement-1.pdf]

bioRxiv preprint doi: https://doi.org/10.1101/2023.01.15.524171; this version posted January 18, 2023. The copyright holder for this preprint (which was not certified by peer review) is the author/funder, who has granted bioRxiv a license to display the preprint in perpetuity. It is made available under aCC-BY 4.0 International license.

SUPPORTING INFORMATION

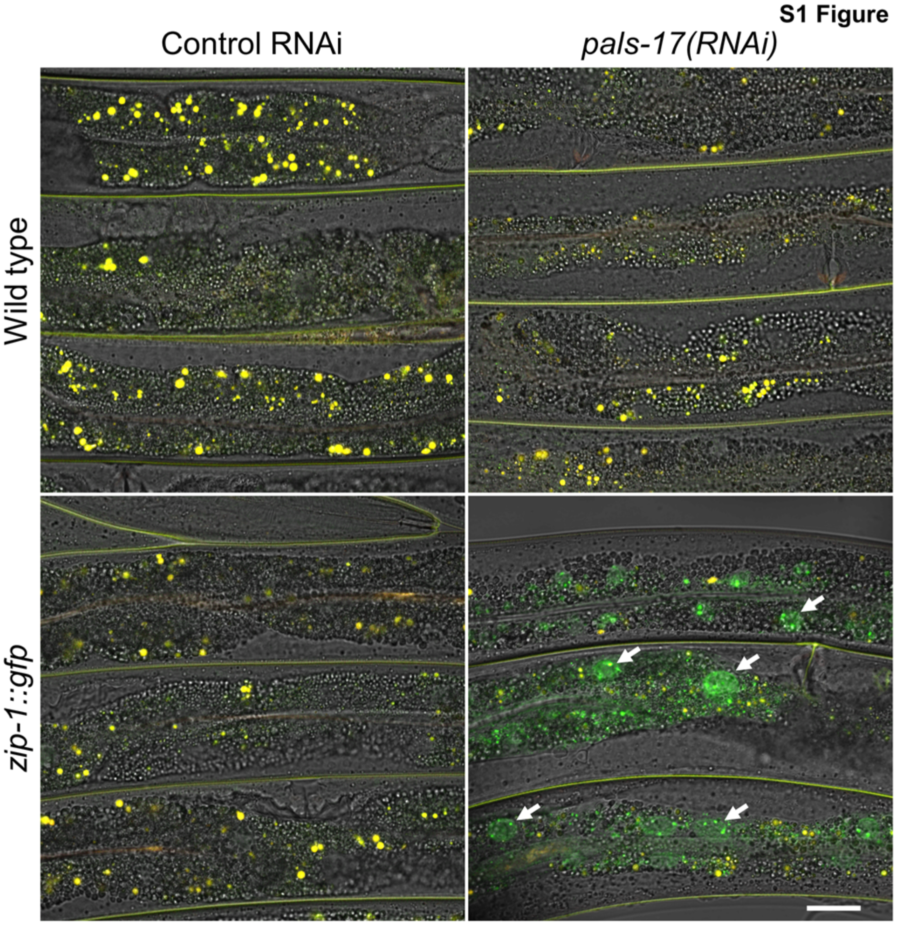

S1 Fig. *pals-17(RNAi)* animals express ZIP-1::GFP in intestinal nuclei

Wild-type and *zip-1::gfp* animals treated with control and *pals-17* RNAi. Green, autofluorescence and DIC channels were merged. Intestinal ZIP-1::GFP expression is indicated with white arrows; autofluorescence from the gut granules and from the cuticle are shown in yellow. Scale bar, 20 µm.

bioRxiv preprint doi: https://doi.org/10.1101/2023.01.15.524171; this version posted January 18, 2023. The copyright holder for this preprint (which was not certified by peer review) is the author/funder, who has granted bioRxiv a license to display the preprint in perpetuity. It is made available under aCC-BY 4.0 International license.

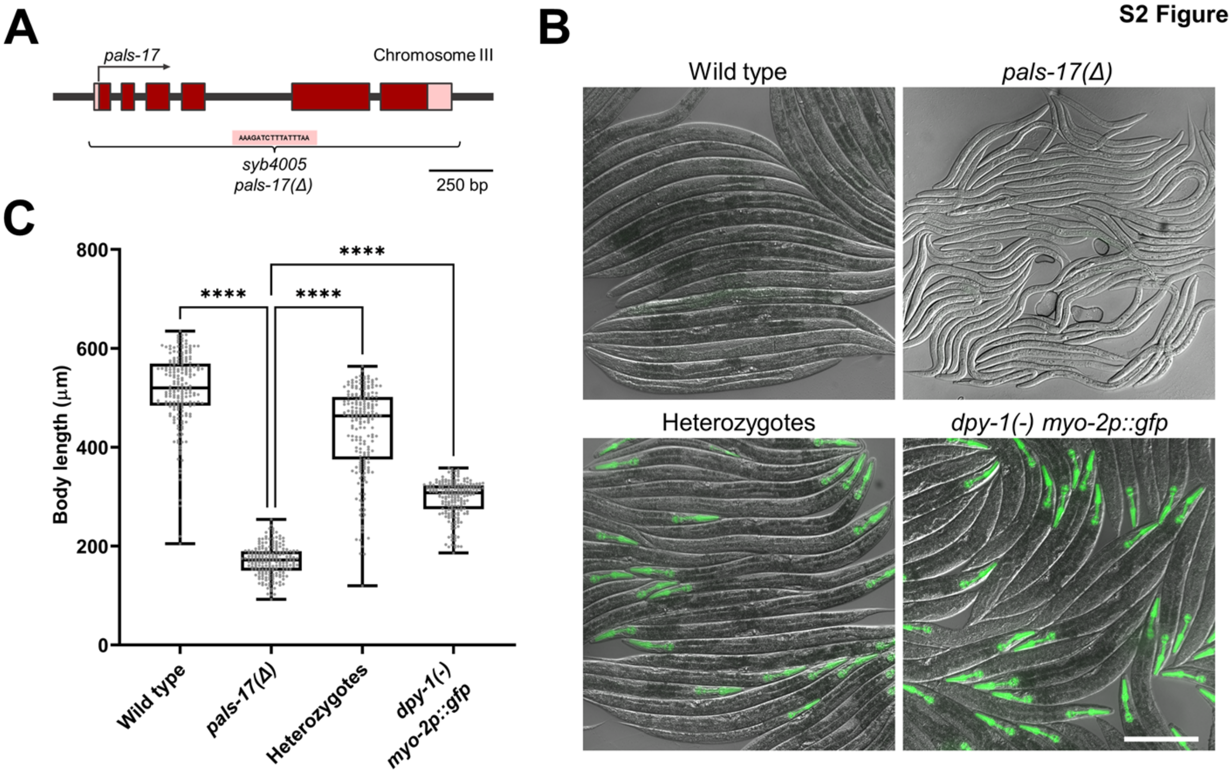

S2 Fig. Deletion of *pals-17* causes developmental arrest

(A) *pals-17* gene structure. Exons are indicated with dark red boxes, 5' and 3' UTRs shown with light red boxes. The deletion in *syb4005* allele (*pals-17(Δ)*) is indicated by the bracket; the inserted sequence is shown in the pink rectangle. The horizontal arrow indicates the direction of transcription. (B) Synchronized *pals-17(Δ)* mutants and control strains following 44 h incubation at 20 °C. Green and DIC channels were merged. *myo-2*p::GFP present in the balancer strain is shown in green. Scale bar, 200 µm. (C) Box-and-whisker plot of body length values for indicated worm strains. Heterozygotes are *pals-17(Δ)/sC1(s2023)*. Box lines represent median values, box bounds indicate 25th and 75th percentiles, and whiskers extend to the minimum and maximum values. Gray dots represent individual values for each animal; 50 animals per each of the three replicates were analyzed. A Kruskal-Wallis test was used to calculate p-values; **** $p < 0.0001$.

bioRxiv preprint doi: https://doi.org/10.1101/2023.01.15.524171; this version posted January 18, 2023. The copyright holder for this preprint (which was not certified by peer review) is the author/funder, who has granted bioRxiv a license to display the preprint in perpetuity. It is made available under aCC-BY 4.0 International license.

S3 Figure

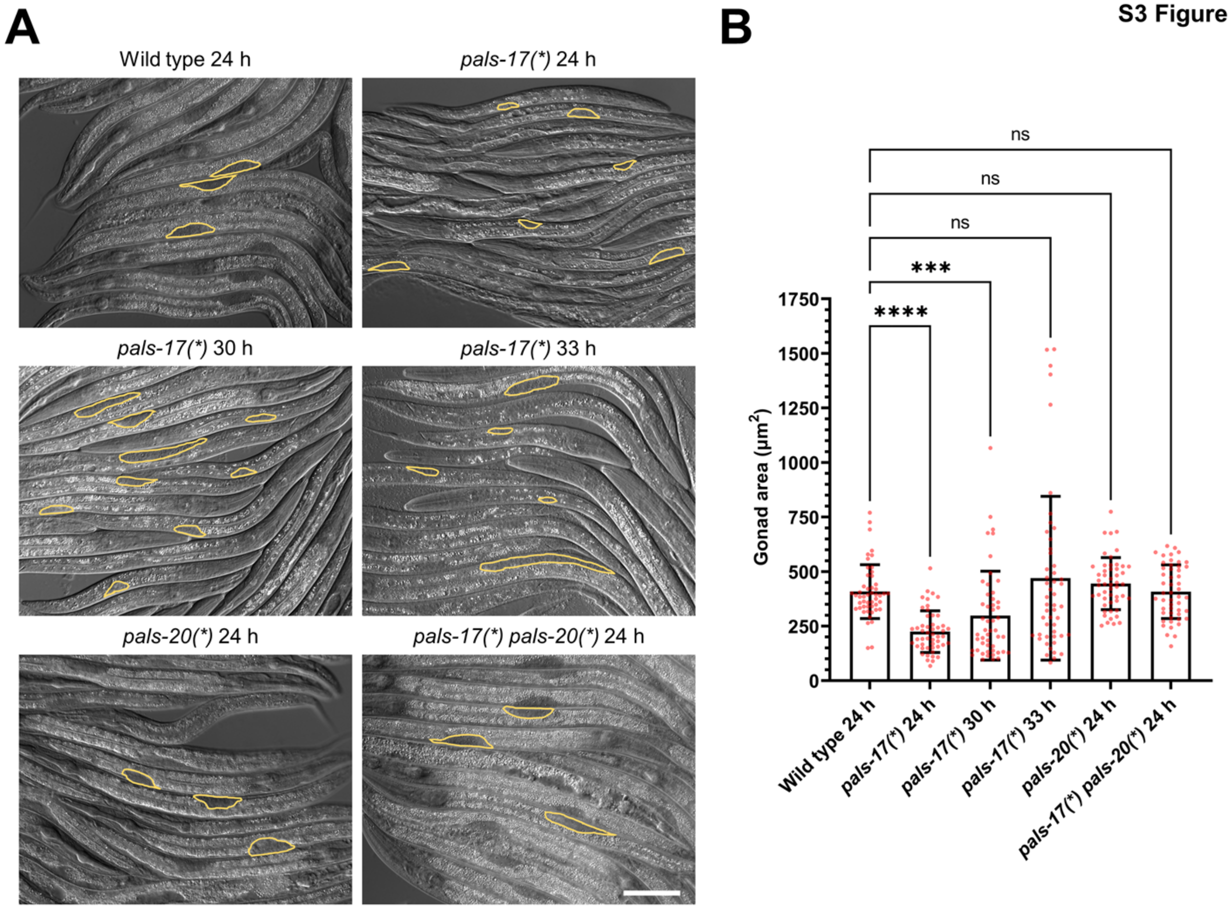

S3 Fig. *pals-17* mutants have asynchronous and delayed development

(A) Representative DIC images of *pals-17* and *pals-20* mutants and wild-type control incubated for 24 h at 20 °C, and images of *pals-17* mutants incubated for 30 h and 33 h at 20 °C from L1 stage. The gonads of some animals are outlined with yellow lines. Scale bar, 60 µm. (B) Gonad area measurements. Results shown are the average of two independent experimental replicates, with 25 animals assayed per replicate. Error bars are SD. A Kruskal-Wallis test was used to calculate p-values; *** $p < 0.001$; **** $p < 0.0001$; ns indicates no significant difference.

bioRxiv preprint doi: https://doi.org/10.1101/2023.01.15.524171; this version posted January 18, 2023. The copyright holder for this preprint (which was not certified by peer review) is the author/funder, who has granted bioRxiv a license to display the preprint in perpetuity. It is made available under aCC-BY 4.0 International license.

S4 Figure

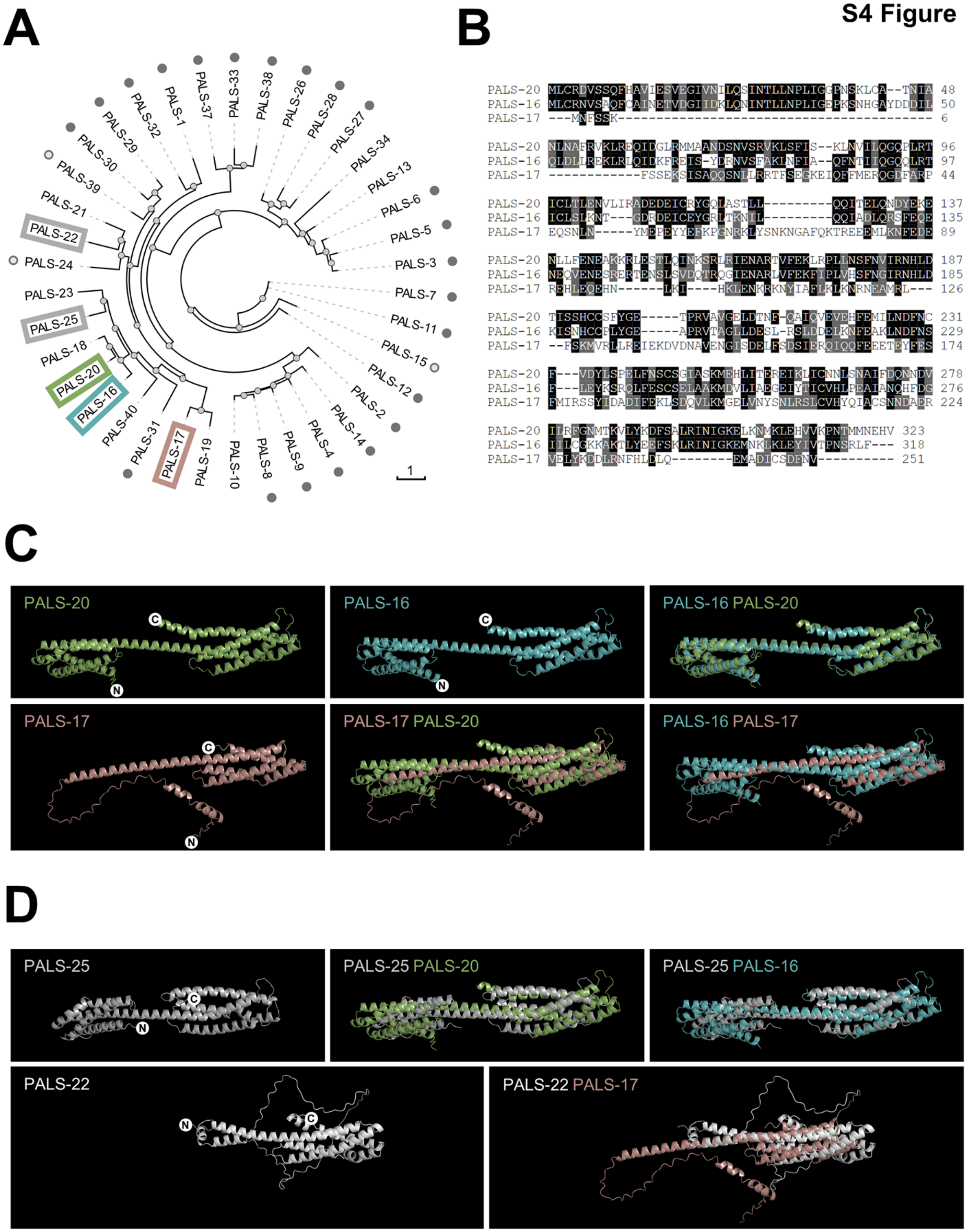

bioRxiv preprint doi: https://doi.org/10.1101/2023.01.15.524171; this version posted January 18, 2023. The copyright holder for this preprint (which was not certified by peer review) is the author/funder, who has granted bioRxiv a license to display the preprint in perpetuity. It is made available under aCC-BY 4.0 International license.

S4 Fig. Amino acid sequence analysis and predicted protein structure analysis of PALS proteins

(A) A radial phylogram of the PALS protein family. Dark gray circles indicate PALS proteins whose corresponding mRNA levels are significantly upregulated following IPR activation during microsporidia infection and in *pals-22* mutants (4, 7). Light grey circles label PALS proteins whose corresponding mRNA levels are significantly upregulated only in *pals-22* mutant background. The branch length is indicated by the scale bar. (B) Amino acid sequence alignment between PALS-20, PALS-16 and PALS-17. Black boxes indicate identical amino acids; grey boxes indicate similar residues (defined in Material and Methods). (C, D) predicted PALS protein structures and their overlap. White circles with letters N and C indicate N- and C-terminuses of PALS proteins, respectively.

bioRxiv preprint doi: https://doi.org/10.1101/2023.01.15.524171; this version posted January 18, 2023. The copyright holder for this preprint (which was not certified by peer review) is the author/funder, who has granted bioRxiv a license to display the preprint in perpetuity. It is made available under aCC-BY 4.0 International license.

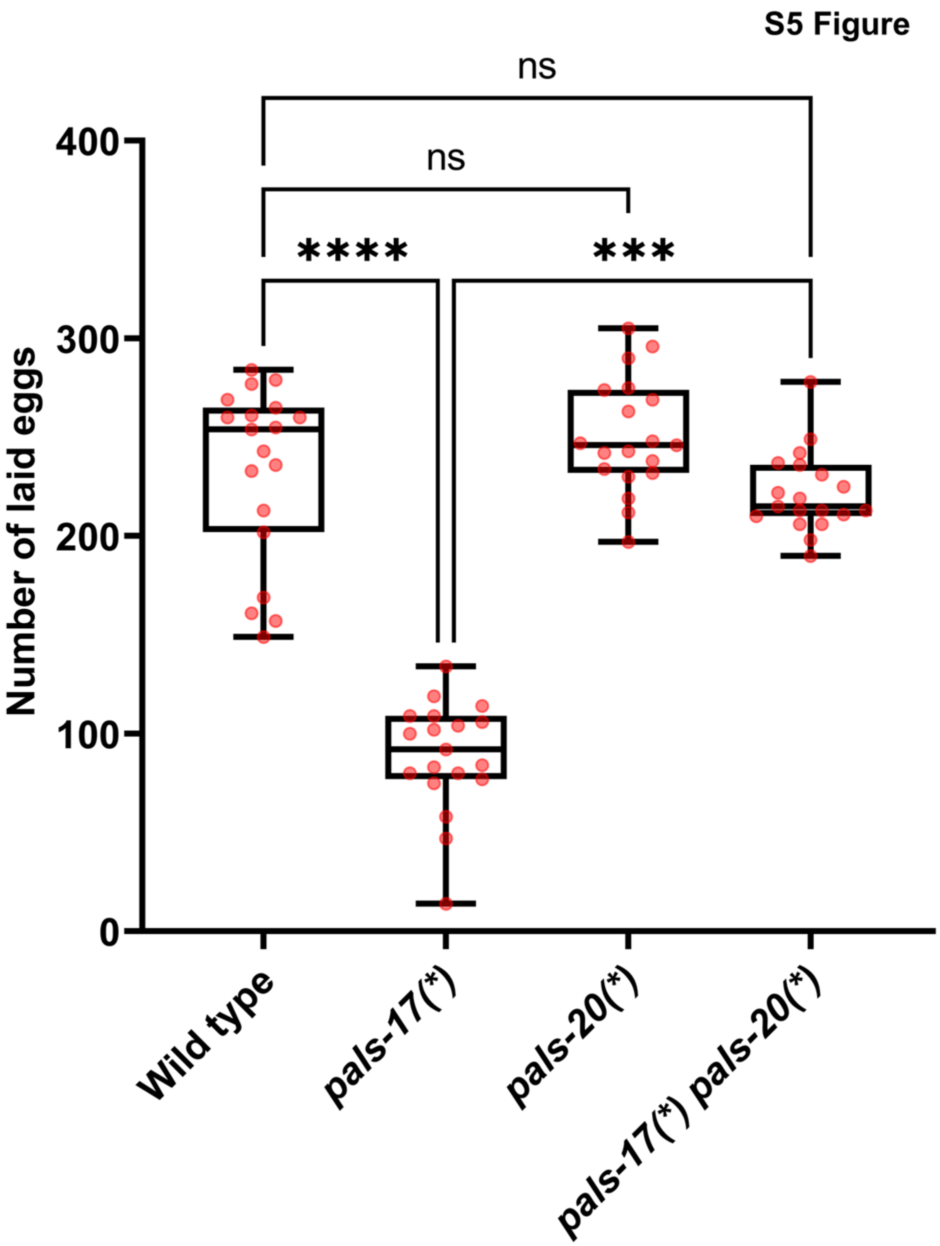

1164

1165 **S5 Fig. *pals-17* and pals-20 regulate brood size of *C. elegans***

1166 *pals-17* mutants have significantly lower brood sizes, and this phenotype is *pals-20*-dependent.
1167 Brood size measurements are shown as a box-and-whisker plot for wild-type, *pals-17(*)*, *pals-*
1168 *20(*)* and *pals-17(*) pals-20(*)* animals. Box lines represent the median values, box bounds
1169 indicate 25th and 75th percentiles, and whiskers extend to the minimum and maximum values. Red
1170 dots represent individual values for each animal; 19 animals were analyzed for each strain (at

bioRxiv preprint doi: https://doi.org/10.1101/2023.01.15.524171; this version posted January 18, 2023. The copyright holder for this preprint (which was not certified by peer review) is the author/funder, who has granted bioRxiv a license to display the preprint in perpetuity. It is made available under aCC-BY 4.0 International license.

least five animals per each of the three experimental replicates). A Kruskal-Wallis test was used to calculate p-values; **** p < 0.0001; *** p < 0.001; ns indicates no significant difference.

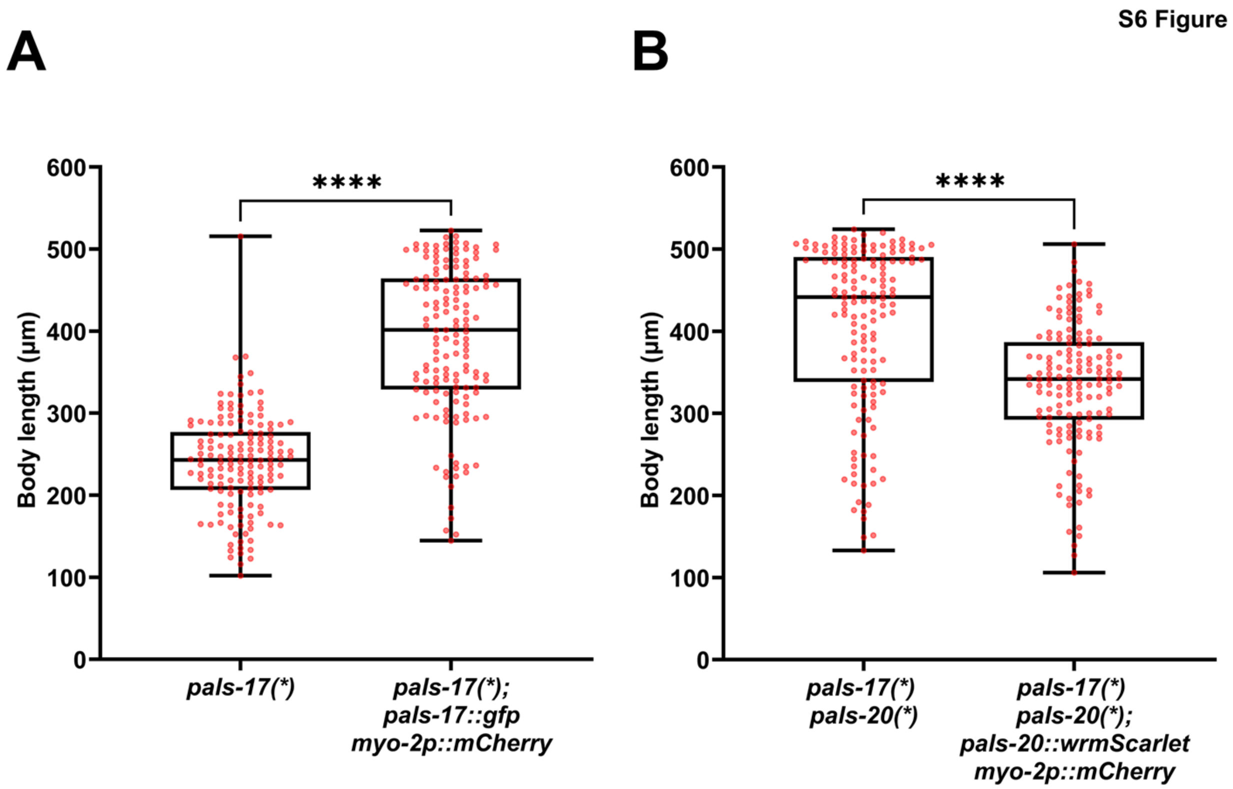

S6 Fig. *pals-17* and *pals-20* translational reporters rescue growth phenotypes of *pals-17(*)* and *pals-17(*) pals-20(*)* mutants, respectively

(A, B) Body length measurements are shown as box-and-whisker plots for *pals-17(*)* (A) and *pals-17(*) pals-20(*)* animals (B). Animals expressing *pals-17::gfp myo-2p::mCherry* (A) and *pals-20::wrmScarlet myo-2p::mCherry* arrays (B) as well as their non-transgenic siblings were analyzed. Box lines represent median values, box bounds indicate 25th and 75th percentiles, and whiskers extend to the minimum and maximum values. Red dots represent individual values for each animal; 50 animals per each of the three experimental replicates were analyzed. A Kolmogorov-Smirnov test was used to calculate p-values; **** p < 0.0001.

bioRxiv preprint doi: https://doi.org/10.1101/2023.01.15.524171; this version posted January 18, 2023. The copyright holder for this preprint (which was not certified by peer review) is the author/funder, who has granted bioRxiv a license to display the preprint in perpetuity. It is made available under aCC-BY 4.0 International license.

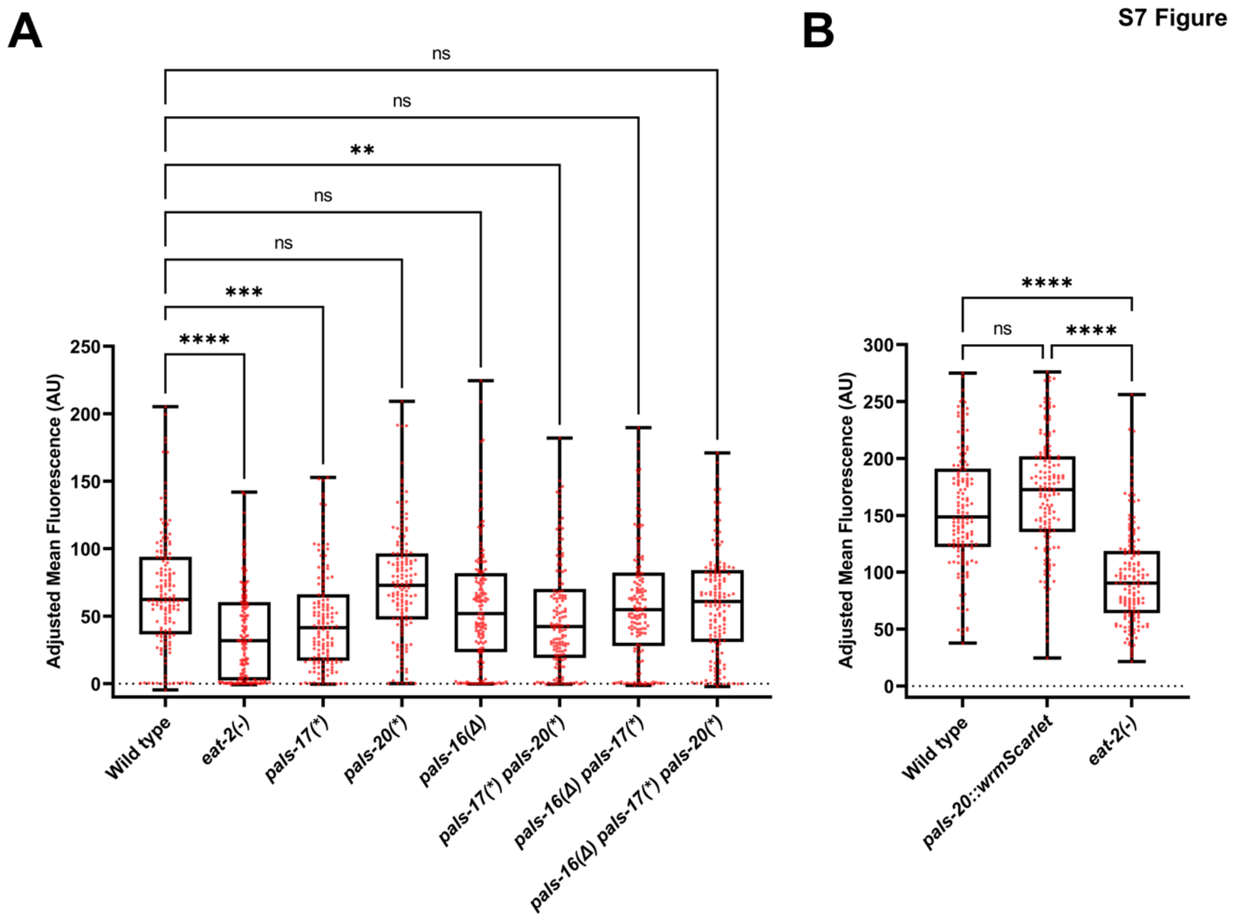

S7 Fig. Quantification of fluorescent bead accumulation in *pals-17*, *pals-20*, *pals-16* mutants and control strains

(A, B) Box-and-whisker plots of bead fluorescence levels per animal. Box lines represent median values, box bounds indicate 25th and 75th percentiles, and whiskers extend to the minimum and maximum values. Data from three independent experimental replicates are shown. Red dots represent individual values for each animal; 50 animals were analyzed per strain per replicate. *eat-2(ad465)* mutant was used as a feeding-defective control. A Kruskal-Wallis test was used to calculate p-values; **** $p < 0.0001$; *** $p < 0.001$; ** $p < 0.01$; ns indicates no significant difference. AU = arbitrary units.

S1 Table. Data shown in graphs in main and supplementary figures

bioRxiv preprint doi: https://doi.org/10.1101/2023.01.15.524171; this version posted January 18, 2023. The copyright holder for this preprint (which was not certified by peer review) is the author/funder, who has granted bioRxiv a license to display the preprint in perpetuity. It is made available under aCC-BY 4.0 International license.

S2 Table. Amino acid sequence analysis of PALS-17, PALS-20 and PALS-16

S3 Table. Differential expression analysis of RNA-seq data

S4 Table. Normalized counts from RNA-seq analyses

S5 Table. Full datasets from GSEA analyses

S6 Table. List of strains used in this study

S7 Table - Primers used in this study
